# Alkali Induction Strategy for Artificial Photosynthesis of Hydrogen by TiO_2_ Heterophase Homojunctions

**DOI:** 10.1002/advs.202413069

**Published:** 2025-02-06

**Authors:** Minghua Xu, Xiaowen Ruan, Malik Zeeshan Shahid, Depeng Meng, Guozhen Fang, Chunsheng Ding, Wei Zhang, Jing Leng, Songcan Wang, Sai Kishore Ravi, Xiaoqiang Cui

**Affiliations:** ^1^ State Key Laboratory of Automotive Simulation and Control School of Materials Science and Engineering, Key Laboratory of Automobile Materials of MOE, Electron Microscopy Center Jilin University Changchun 130012 China; ^2^ School of Energy and Environment City University of Hong Kong Tat Chee Avenue Kowloon Hong Kong SAR 999077 China; ^3^ State Key Laboratory of Molecular Reaction Dynamics, Dalian Institute of Chemical Physics Chinese Academy of Sciences Dalian 116023 China; ^4^ Frontiers Science Center for Flexible Electronics, Xi'an Institute of Flexible Electronics (IFE) Northwestern Polytechnical University Xi'an 710072 China

**Keywords:** charge carrier kinetics, homojunction structure, heterophase, hydrogen evolution, redox‐active sites

## Abstract

The robust separation and utilization of photogenerated electrons‐holes (e^−^‐h^+^) are key in accelerating redox reactions. Unlike traditional heterojunction photocatalysts, homojunction features different energy bandgaps with interchangeable compositions that can significantly trigger charge carrier dynamics, but their precise construction remains an ongoing challenge owing to quick lattice‐level modulations. Herein, TiO_2_‐based homojunction (HT_M‐OH_) holding dissimilar yet discernible crystalline phases (anatase and rutile) are rationally constructed by a straightforward alkali‐induced strategy which enables controllable lattice‐transition/orientation. The resulting HT_M‐OH_ exhibits speedy separation and well‐guided flow of e^−^‐h^+^ over redox sites with extended carrier lifetime, leading to high‐rate hydrogen generation (HER, 34.35 mmol g^−1^ h^−1^) under simulated sunlight. Moreover, a self‐made thin film of HT_M‐OH_ indicates a notable scale‐up potential under real‐time sunlight. This work furnishes a new non‐complex homojunction strategy for speeding charge carrier kinetics, credibly extendable to a diverse range of catalysts and applications.

## Graphical abstract

A straightforward alkali‐induced strategy is implemented to rationally construct controllable lattice‐transition/orientation in TiO_2_‐based homojunction (HTM‐OH). The resulting HTM‐OH exhibits speedy separation and well‐guided flow of e^−^‐h^+^ over redox sites with extended carrier lifetime, leading to high‐rate hydrogen generation (HER, 34.35 mmol g^−1^ h^−1^) under simulated sunlight and a notable scale‐up potential under real‐time sunlight.

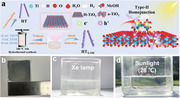

## 1 Introduction

Solar‐driven water splitting into clean hydrogen fuel (i.e., HER) is a potential long‐term, sustainable and green strategy to alleviate the energy crisis.^[^ ^1^, ^2^ ^]^ Up to now, a series of photocatalysts have been investigated, such as organic framework materials, metal oxides, sulfides, and molecular materials. Given that TiO_2_ has shown outstanding stability and a nontoxic nature, it has become one of the most widely studied photocatalysts. However, the intrinsically wide band gap (≈3.2 eV) limits its absorption in the visible region and the unavoidable recombination of photogenerated charge carriers significantly hinders redox reaction processes.^[^ ^3^, ^4^ ^]^ So, improving the light‐absorption range and charge carrier separation efficiency of TiO_2_‐based photocatalysts is the key to achieving optimum photocatalytic performance.

Photocatalytic homojunction, composed of semiconductors with analogous composition, is considered a potential way to address issues of photogenerated charge carrier recombination.^[^ ^5^, ^6^ ^]^ Owing to their properties of faster carrier transfer through chemically similar interfaces, they have received wide attention recently. For example, homojunction of carbon nitride, MOF, perovskite, and sulfide materials have been developed to achieve improved performance.^[^ ^7^, ^8^, ^9^, ^10^ ^]^ However, unlike the complex traditional strategies, creating a non‐complex and controllable approach for realizing precise homojunction (particularly TiO_2_‐based) with different phases is challenging. This is because homojunction generally originates from the abrupt self‐transitions and orientations of atoms during the nucleation and growth of a photocatalyst. Particularly, it is even more intriguing to induce such discernible phases that enable quick charge separation with less probability of recombination and allow the directed flow of e^−^‐h^+^ over redox sites with extended carrier lifetime, thereby efficiently driving redox reactions.^[^ ^11^, ^12^ ^]^ Besides, developing a low‐cost TiO_2_‐based homojunction photocatalyst with scale‐up potential is a highly desirable target.

Herein, we introduce a new non‐complex and robust alkali‐induced strategy (using NaOH instead of the traditional addition of tetrabutyl titanate (TBOT)) which allows pH modulation, thereby controlling the sudden atomic‐level transitions to realize TiO_2_‐based homojunction (HT_M‐OH_). Since OH^−^ can be regarded as coordinating agents for metal ions to vary their mass transport rates, the obtained rich OH− environment can lead to the mass‐transport limitation for metal ions. In this mass transport limited process, any thermodynamic fluctuations could lead to the nucleation of twin planes due to their low formation energy. The intimate contact of heterophases (anatase and rutile) in HT_M‐OH_ enables type‐II homojunction, allowing accelerated spatial charge separation with significantly reduced recombination and directing the settling of long‐life e^−^‐h^+^ over redox sites. Consequently, HT_M‐OH_ exhibits high‐rate HER (34.35 mmol g^−1^ h^−1^) under simulated irradiation, while a flexible HT_M‐OH_ film (loaded 1.0 wt% Pt co‐catalyst) manifests a scale‐up potential under real‐time sunlight. This work offers a novel strategy for triggering charge carrier dynamics and provides an in‐depth understanding applicable to advancing diverse catalysts for energy‐related applications.

## 2 Results and Discussion

### 2.1 Morphology and Structure

**Figure 1:**
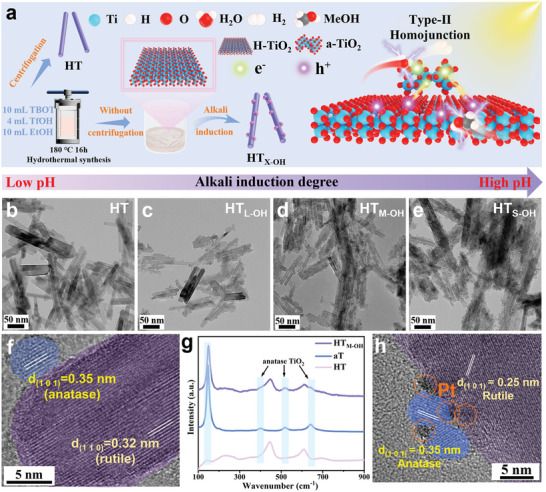
a) The schematic diagram of TiO_2_ homojunction synthesis and electron transfer mechanism. b–e) TEM images of HT and HT_X‐OH_ (L, M, and S‐OH represent low, medium, and superfluous OH concentrations in reaction solution respectively). f) HRTEM image of HT_M‐OH_. g) Raman spectra of HT, aT and HT_M‐OH_. h) HRTEM image of HT_M‐OH_ with Pt loading.

The TiO_2_ heterophase homojunction photocatalyst is synthesized using a mild alkali‐induction method (**Figure** **1**a). First, pristine H‐doping titanium dioxide (HT) is synthesized by hydrothermal synthesis using TBOT, trifluoromethanesulfonic acid (TfOH) and absolute ethanol (EtOH) according to our previous research work. For previous work, we obtained pristine HT after washing and centrifuging the acid reaction mixture. Now, we realize that TiO_2_ heterophase homojunction photocatalysts (HT_X‐OH_) can be obtained directly without the additional addition of TBOT via alkali induction treatment of reaction mixture, and form a carrier transport mechanism satisfying the type‐II structure.

Transmission electron microscope (TEM) results show that there are more and more nanoparticles loaded on the nanorods with increasing the pH of the acid reaction mixture (Figure 1b–e). The structure is further investigated using high‐resolution TEM (HRTEM), as shown in Figure 1f. The lattice spacings of 0.32 nm and 0.35 nm are in good agreement with the (110) and (101) planes of rutile and anatase phase of TiO_2_, respectively.^[^ ^13^ ^]^ Figure 1g illustrates that the three new observed peaks at 401, 518 and 641 cm^−1^ are attributed to the modes of anatase phase of TiO_2_ (aT) in as‐prepared HT_M‐OH_ catalyst in comparison with pristine HT.^[^ ^14^ ^]^ The main structure of HT is well retained in the formed heterophase homojunction materials proved by XRD patterns and the corresponding energy dispersive X‐ray spectroscopy (EDS) (Figures S1,S2 of the Supporting Information). Meanwhile, the (110) and (101) planes of rutile and anatase phase observed in HRTEM is well matched with the diffraction peaks at 27.4° and 25.3° detected in XRD patterns. All these results demonstrate that the heterophase homojunction is successfully synthesized. We also observed that the co‐catalyst Pt tended to aggregate on aT after the photocatalytic evolution reaction (Figure 1h).^[^ ^15^ ^]^ This implies that aT is the main reduction site during the photocatalytic reaction and the additional evidence will be discussed in the subsequent description.

### 2.2 Catalyst Composition and Photogenerated Carrier Dynamics

**Figure 2:**
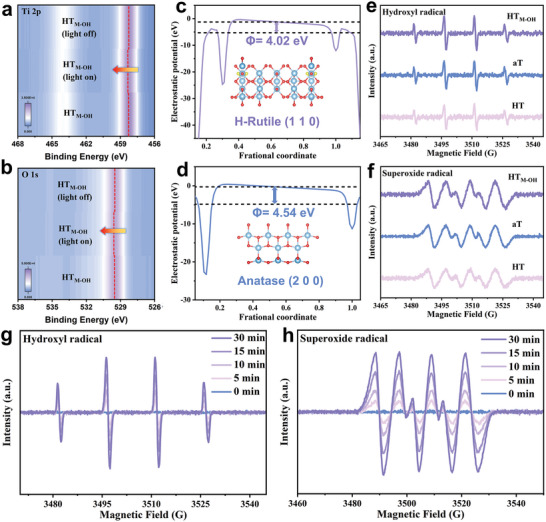
In situ X‐ray photoelectron spectroscopy spectra of a) Ti 2p, b) O 1s. c,d) The work functions of HT and aT, respectively. e) in aqueous dispersion for DMPO‐hydroxyl radical under UV–vis light for 5 min and f) in methanol dispersion for DMPO‐superoxide radical, respectively. In situ ESR spectra of HT_M‐OH_ g) aqueous dispersion and h) in methanol dispersion under 0, 5, 10, 15, and 30 min of light irradiation, respectively.

To further confirm the mechanism of spatially charge separation, in situ irradiated X‐ray photoelectron spectroscopy (XPS) spectra are used to explore the charge transfer at the interface of TiO_2_ heterophase homojunction (**Figure** **2**a,b). The Ti 2p XPS spectra are fitted to two peaks at 458.2 and 464.0 eV, which can be attributed to Ti 2p_3/2_ and Ti 2p_1/2_ of Ti^4+^, respectively. The high‐resolution O 1s spectra are fitted to two peaks at 529.5 and 530.7 eV, which can be attributed to Ti‐O and adsorbed OH^−^ species.^[^ ^16^ ^]^ When HT_M‐OH_ is exposed under illumination, both the Ti 2p and the O 1s peaks shift to higher binding energy (Figure 2a,b; Figures S3,S4 of the Supporting Information). This suggests the decreased electron density around HT and increased electron density on aT, respectively, which suggests that photogenerated electrons in HT migrate to aT at the interfaces under light irradiation.^[^ ^17^, ^18^ ^]^

Based on the electrostatic potential, the energy difference between the vacuum and Fermi levels can be estimated. We calculated the theoretical work functions of HT (110) and aT (200) to be 4.02 and 4.54 eV, respectively (Figure 2c,d).^[^ ^19^ ^]^ When HT and aT come into contact, the free electrons migrate from HT to aT through the intimate interface until their Fermi levels are equilibrated, which is consistent with the electron flow probed by XPS analysis. To further study the charge transfer in the TiO_2_ heterophase homojunction, electron spin resonance (ESR) spin‐trap experiments are conducted. The results demonstrate that the ESR signals of superoxide radicals and hydroxyl radicals are not significantly changed among samples of HT, aT and HT_M‐OH_ (Figure 2e,f). These results mean that a Type‐II heterojunction in HT_M‐OH_ is successfully formed. The intensity of both free radicals exhibits a continuously increasing trend under light exposure, while no active radicals are detected in dark conditions (Figure 2g,h; Figures S5,S6 of the Supporting Information). This suggests that the heterophase homojunction structure can continuously and efficiently generate reactive radicals for photocatalytic reactions.

**Figure 3:**
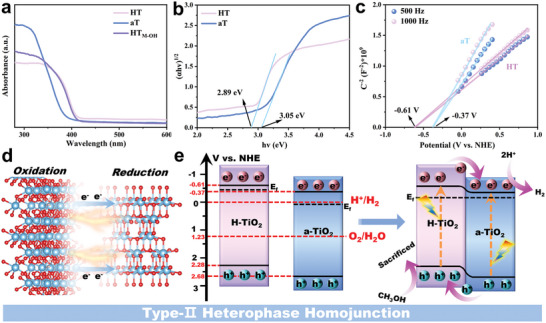
a) UV–vis spectra. b) Tauc plots. c) Mott‐Schottky plots of HT and aT catalysts. d) Illustration of carrier transfer between heterophase homojunction. e) Schematic diagram of band structure.

The band structures of the HT_M‐OH_ heterophase homojunction structure are determined using ultraviolet−visible (UV–vis) spectra and Mott–Schottky (M–S) plots. As shown in **Figure** **3**a, all samples exhibited intense absorption in the UV region. After HT and aT heterophase homojunction is formed, the absorbing edge of HT_M‐OH_ is not shifted. The results indicate that the constructed homojunction catalysts have no significant change for the bandgaps. The bandgaps of HT and aT are estimated as 2.89 and 3.05 eV from the Tauc plots in Figure 3b (the curve of converted (αhv)^1/2^ versus hv from the UV–vis spectrum, where α is the absorption coefficient, h is the Planck's constant, v is the light frequency).^[^ ^20^ ^]^ M‐S plots test is often used to estimate conduction band potentials of semiconductors.^[^ ^21^, ^22^ ^]^ The results all exhibit positive slopes, implying the n‐type‐semiconducting nature of the electronic band structures, and reveal the conduction band potentials of HT and aT to be approximately ‐0.61 V and ‐0.37 V (versus NHE), respectively (Figure 3c). According to the equation E_CB_ + E_g_ = E_VB_, the valence band potentials could be calculated as 2.28 V and 2.68 V for HT and aT. Due to appropriate energy band alignment and iso‐elemental lattice matching, they can be combined more easily and robustly (Figure 3d). Taking the above results together, we provide the schematic diagram of band structure, showing that the Type‐II heterojunction structure can efficiently achieve the charge spatial separation and improve the photocatalytic hydrogen evolution reaction under light illumination (Figure 3e).

### 2.3 Charge Transportation Property

**Figure 4:**
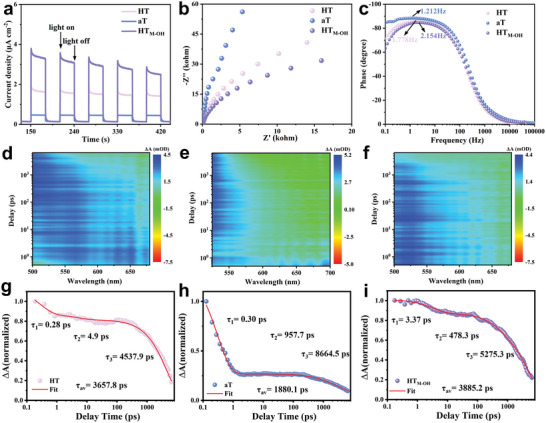
a) Transient photocurrent density, b) Electrochemical impedance spectroscopy (EIS) Nyquist plots and c) Bode phase spectrum of aT, HT and HT_M‐OH_ catalyst. Transient absorption spectra of d,) HT, e) aT and f) HT_M‐OH_ catalyst. Normalized transient absorption kinetics probed at 600 nm,600 nm and 608 nm for g) HT, h) aT and i) HT_M‐OH_ catalyst after 400 nm laser excitation, respectively.

Several photoelectrochemical (PEC) measurements are further carried out to study the charge transfer dynamics and mechanism. As shown in **Figure** **4**a–c, under light irradiation, HT_M‐OH_ shows a significantly higher photocurrent density, a smaller radius of the Nyquist plots and a larger peak frequency at minimum phase difference.^[^ ^23^, ^24^ ^]^ It can be attributed to intense interactions at interfaces of heterophase homojunction, which promotes charge carrier separation and migration efficiency. Several further characterizations of photoluminescence spectra and time‐resolved fluorescence spectra further confirm the efficient separation and transportation of photogenerated carriers in HT_M‐OH_ catalyst (Figures S7,S8 of the Supporting Information).

To further explore the charge transfer kinetics and mechanisms, the femtoseond transient absorption (fs‐TA) spectra of HT, aT and HT_M‐OH_ catalysts are collected. The excitation wavelengths for all catalysts are set at >400 nm based on the corresponding band structures. Upon photoexcitation, the results demonstrate that HT and HT_M‐OH_ catalysts show broadband absorption within the probe light range of 500–650 nm, while the aT catalysts only show absorption within the probe light range of 500–575 nm. (Figure 4d–f; Figures S9–S11 of the Supporting Information). To investigate the lifetime of the carriers in as‐prepared catalysts, we fitted the TA kinetic curves by the three‐exponential function (Figure 4g–i). HT_M‐OH_ catalysts exhibit a longer active charge complexation process and the lifetime increases to 3885.2 ps (Table S1, Supporting Information). The results suggest that there is a high efficiency of charge separation and transfer of the photogenerated electron‐hole pairs in the heterophase homojunction structure, and the charge self‐trapping process is suppressed.^[^ ^25^, ^26^ ^]^ The kinetic analysis above indirectly authenticates the accelerated charge separation in constructed heterophase homojunction again.

### 2.4 Photocatalytic Hydrogen Evolution Performance of the As‐Prepared Photocatalyst

**Figure 5:**
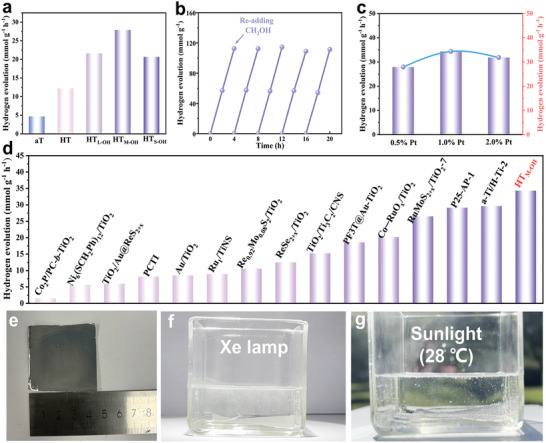
a) Photocatalytic hydrogen evolution over time for aT, HT and HT_X‐OH_. b) Cycling experiments of HT_M‐OH_. c) HT_M‐OH_ with different Pt loading. d) Photocatalytic hydrogen evolution rate of HT_M‐OH_ compared with previous TiO_2_‐based photocatalysts.^[^ ^14^, ^27^, ^28^, ^29^, ^30^, ^31^, ^32^, ^33^, ^34^, ^35^, ^36^, ^37^, ^38^, ^39^ ^]^ e) Flexible photocatalyst films. The evolution state of the photocatalyst film in f) Xe lamp light and g) natural sunlight conditions.

UV‐vis light‐driven HER activities of the above catalysts are evaluated and displayed in **Figure** **5**a. The hydrogen evolution rates of the pristine aT and HT are 4.70 and 12.20 mmol g^−1^ h^−1^, and HT_M‐OH_ catalysts show an increased hydrogen evolution performance to 27.91 mmol g^−1^ h^−1^. The stability of the HT_M‐OH_ catalysts is also assessed through long‐term cycling experiments and the results confirmed its superior stability, which was further corroborated by XRD patterns, XPS data and SEM image taken after the photocatalytic reaction (Figure 5b; Figures S12–S14 of the Supporting Information). Additionally, to optimize performance, various loadings of platinum (Pt) on the HT_M‐OH_ catalysts are investigated, a loading of 1.0 wt% Pt on HT_M‐OH_ shows the highest rate in all samples at 34.35 mmol g^−1^ h^−1^, which is around 7.31‐fold and 2.82‐fold increase compared to that of pristine aT and HT (Figure 5c). Moreover, HT_M‐OH_ shows the apparent quantum efficiency (18.1%) at 380 nm (Figure S15 of the Supporting Information). Compared with other reported TiO_2_‐based photocatalysts, this obtained performance shows an extraordinary hydrogen evolution (Figure 5d and Table S2, Supporting Information).

For possible scale‐up applications, particulate photocatalysis also meet the issues in terms of the separation and recycle of the photocatalysts. We also prepared a flexible HT_M‐OH_ loaded 1.0 wt% Pt co‐catalyst films with dimension of 4.5 cm × 4.5 cm on aluminum foil (Figure 5e–g). Impressively, both under Xe lamp light and natural sunlight, the film can generate observable hydrogen gas bubbles, implying the potential for possible scale‐up solar‐driven H_2_ production (Videos S1,S2, Supporting Information).

## 3 Conclusion

In conclusion, we report a non‐complex alkali‐induced strategy to build TiO_2_‐based homojunction catalysts on the substrate of HT. The tight combination of HT and aT can be observed in the constructed homojunction catalysts. In situ XPS and other characterizations verify that their matched band structures formed a type‐II heterostructure, effectively promoting photogenerated carrier separation. The carrier lifetime of the obtained catalysts is improved under the observation of fs‐TA spectroscopy. The resulting optimized catalyst exhibits an excellent photocatalytic hydrogen evolution rate of 34.35 mmol g^−1^ h^−1^ under UV–vis light irradiation. Furthermore, the prepared films coated with catalysts show a large number of bubbles observed under simulated and natural sunlight. This research provides an alkali‐induced strategy for the construction of robust homojunction structure catalysts to improve photogenerated carrier transport and separation efficiency enhancing photocatalytic hydrogen evolution reaction performance.

## Conflict of Interest

The authors declare no conflict of interest.

## Supporting information



Supporting Information

Supplemental Video 1

Supplemental Video 2

## Data Availability

Research data are not shared.
