# Supplementary material for: Alkali Induction Strategy for Artificial Photosynthesis of Hydrogen by TiO2 Heterophase Homojunctions
Source: Adv Sci (Weinh). 2025 Feb 6;12(12):2413069. doi: 10.1002/advs.202413069 (PMC11948080; doi:10.1002/advs.202413069)
Supplement: Supplementary file 1 — Supporting Information [file ADVS-12-2413069-s002.pdf]

ADVANCED SCIENCE
Open Access

Supporting Information

for *Adv. Sci.*, DOI 10.1002/advs.202413069

Alkali Induction Strategy for Artificial Photosynthesis of Hydrogen by TiO_2 Heterophase Homojunctions

Minghua Xu, Xiaowen Ruan, Malik Zeeshan Shahid, Depeng Meng, Guozhen Fang, Chunsheng Ding, Wei Zhang, Jing Leng, Songcan Wang, Sai Kishore Ravi* and Xiaoqiang Cui**

Alkali Induction Strategy for Artificial Photosynthesis of Hydrogen by TiO_2 Heterophase Homojunctions

Minghua Xu,[1] Xiaowen Ruan,*[2] Malik Zeeshan Shahid,[1] Depeng Meng,[1] Guozhen Fang,[1] Chunsheng Ding,[1] Wei Zhang,[1] Jing Leng,[3] Songcan Wang,[4] Sai Kishore Ravi,*[2] and Xiaoqiang Cui*[1]

[1] State Key Laboratory of Automotive Simulation and Control, School of Materials Science and Engineering, Key Laboratory of Automobile Materials of MOE, Electron Microscopy Center, Jilin University, Changchun 130012, China

[2] School of Energy and Environment, City University of Hong Kong, Tat Chee Avenue, Kowloon, Hong Kong SAR

[3] State Key Laboratory of Molecular Reaction Dynamics, Dalian Institute of Chemical Physics, Chinese Academy of Sciences, Dalian 116023, China

[4] Frontiers Science Center for Flexible Electronics, Xi'an Institute of Flexible Electronics (IFE), Northwestern Polytechnical University, 127 West Youyi Road, Xi'an, 710072, China

*Corresponding Author: Xiaoqiang Cui; Xiaowen Ruan; Sai Kishore Ravi

E-mail: xqcui@jlu.edu.cn (X. Q. Cui); xiaoruan@cityu.edu.hk; skravi@cityu.edu.hk

Experimental section

1. Materials ··········· 03

2. Synthesis of HT, aT and HT_{X-OH} samples ··········· 03

3. Preparation of the film ··········· 03

4. Characterization··········· 04

5. Photocatalytic activity ··········· 04

6. Photoelectrochemical measurement··········· 04

7. DFT calculations··········· 05

8. Ultrafast TA spectroscopy Measurement ··········· 05

Supplemental Figures and Table

1. Figure S1-S15 ··········· 07-21

2. Table S1 ··········· 22

3. Table S2 ··········· 23

References

1. References ··········· 24

Experimental section

1. Materials

Deionized water (DI water, 18.2 MΩ cm) is employed throughout the experiments. All chemicals are analytical grade and used as received without further purification. Tetra-butyl (IV) titanate (TBOT), anhydrous ethanol (C_2H_5OH) sodium hydroxide (NaOH) and methanol (MeOH) purchased at Sinopharm Chemical Reagent Co, Ltd (China). Trifluoromethanesulfonic acid (TfOH) and chloroplatinic acid hexahydrate are purchased from Sigma-Aldrich. Poly (vinylidene fluoride) (average Mw ~ 400,000 pellets) and 1-Methyl-2-pyrrolidinone are purchased from Macklin.

2. Synthesis of HT, aT and $HT_{X\text{-}OH}$ samples

In the typical synthesis, for the preparation of HT and $HT_{M\text{-}OH}$, 4 mL of solution of TfOH was cautiously added dropwise to 10 mL of TBOT in a polytetrafluoroethylene reactor (50 mL) under continuous stirring. Then, 10 mL of ethanol was added to the above mixture. The mixture in the reactor was put into a Teflon-lined stainless autoclave at 180 °C for 16 h. The H-TiO_2 solution obtained after high temperature reaction was divided into four parts and placed in 100 mL beakers. 20 mL of ethanol were added to the beakers and stirred for 10 minutes. After that 5 M NaOH solution was used to adjust the pH of the three solution to 5, 13, 14 and stirred for several hours. The final product was collected by centrifugation washing with DI water and ethanol three times, and then dried in vacuum oven at 60 °C for 8 h (labeled as HT, $HT_{L\text{-}OH}$, $HT_{M\text{-}OH}$ and $HT_{S\text{-}OH}$). For the synthesis of pristine aT, the procedure is the same as HT except the fact that solution of TfOH is replaced with distilled water.

3. Preparation of the film

First, $HT_{M\text{-}OH}$ photocatalyst is homogeneously dispersed in 80 mL of water and 20 ml of methanol solution. H_2PtCl_6 is used to provide 1 wt% Pt. Vacuum and 5℃ are maintained during the experiment. The Pt co-catalysts are loaded on the photocatalysts under 30 min illumination. And then 150 mg of the obtained catalysts and 50 mg Poly (vinylidene fluoride) powder are added into a mortar, Then, 2 mL of 1-Methyl-2-pyrrolidinone as the solvent is mixed in the above mixture and ground thoroughly for

30 minutes to obtain a slurry. An appropriate amount of the slurry is placed on aluminum foils with dimensions of 4.5 × 4.5 cm, and the obtained films are then dried in a vacuum oven at 60 °C for 1 h.

4. Characterization

The crystal structure is revealed by the powder X-ray diffraction (XRD) on a Bragg-Brentano diffractometer (D8-tools, Germany) equipped with a Cu Kα source. Carry out XPS to investigate the chemical states of the elements on the ESCALAB-250Xi instrument (Thermo Fisher Scientific, USA). In-situ XPS is conducted under the same condition, except that UV–visible-light irradiation is introduced. Use the JEM-2100F transmission electron microscope (JEOL CO., Japan) to collect the transmission electron microscope (TEM) and energy-dispersive X-ray spectroscopy (EDX) under the condition of 200 kV accelerating electric field, and the morphology of the samples are from a field emission scanning electron microscope (FESEM) (Hitachi, SU8010, Japan). The Raman spectra was obtained by Horiba HR Evolution. For this a 532 nm laser served as the excitation source. The ultraviolet-visible (UV-Vis) spectra are measured with a UV-Vis spectrophotometer (Shimadzu, Japan, UV2550). Electron spin resonance (ESR) analysis is performed using a Jeol/JES-FA200 to study the mechanism of the photocatalysts. For ESR analysis, 50 μL of DMPO (5,5-dimethyl-1-pyrroline N-oxide) with 10 mg of photocatalyst is added to 0.5 mL of distilled water/methanol. The transient photocurrent response and electrochemical impedance spectra (EIS) analysis were determined by using a CHI760E electrochemical workstation.

5. Photocatalytic activity

Without particular instructions, 10 mg of catalyst is dispersed in a mixture of 80 mL of water and 20 ml of MeOH, and used H_2PtCl_6 to provide 0.5 wt% Pt. Vacuum and 5°C are maintained during the experiment. Provide simulating sunlight conditions using a 300 W Xe lamp with a filter (λ > 350 nm). The GC2014C gas chromatography system is equipped with a thermal conductivity detector, using Ar as the carrier gas and a 5Å molecular sieve column to record the photocatalytic hydrogen production reaction.

6. Photoelectrochemical measurement

Dissolved the sample in 0.5 vol% Nafion-Isopropanol solution and dispersed it on

FTO conductive glass as a photoelectrode. 0.5 M Na_2SO_4 solution was used as the electrolyte. The working electrode, counter electrode and reference electrode are FTO glass carrying the sample, a Pt foil and an Ag/AgCl (saturated KCl), respectively.

7. DFT calculations

All spin-olarized density functional theory (DFT) calculations were performed with the plane-ave basis set as implemented in the Vienna Ab Initio Simulation Package (VASP)[1, 2], and the electrons and ions interactions were described by the projector augmented wave (PAW) potential[3, 4]. The exchange–correlation interactions were determined by the Perdew–Burke–Ernzerhof (PBE) functional within the generalized gradient approximation (GGA)[5] .The plane wave energy cutoff of 500 eV, and the convergence criterion for the residual force and energy was set to 0.05 eV $Å^{-1}$ and 10^{-5} eV, respectively. The empirical correction in Grimme's method (DFT+D3) was used to describe the van der Waals (vdW) interactions[6]. The Brillouin region was sampled by the Monkhorst-Pack method with a 3× 3 × 1 k-point mesh. A vacuum of 15Å was introduced to minimize interactions between adjacent layers in supercells. For the calculation of the work function, the following expression, $\varphi=E_{vac}-E_F$ is used, where E_{Vac} is the electrostatic potential energy of the supercell in the vacuum region, and E_F is the Fermi energy level.

8. Ultrafast TA spectroscopy Measurement

The femtosecond transient absorption setup is based on a regenerative amplified Ti:sapphire laser system from Coherent (800 nm, 35 fs, 6 mJ $pulse^{-1}$, and 1 kHz repetition rate), nonlinear frequency mixing techniques and the Femto-TA100 spectrometer (Time-Tech Spectra LLC). Briefly, the 800 nm output pulse from the regenerative amplifier was split in two parts with a beam splitter. The transmitted part was used to pump a β-BaB_2O_4 crystal (BBO) which generates a 400 nm pump beam. The reflected part with less than 10% was attenuated with a neutral density filter and focused into a 2 mm thick CaF_2 window to generate a white light continuum (WLC) from 340 nm to 750 nm used for probe beam. The probe beam was focused with an Al parabolic reflector onto the sample. After the sample, the probe beam was collimated

and then focused into a fiber-coupled spectrometer with CMOS sensors and detected at a frequency of 1 kHz. The delay between the pump and probe pulses was controlled by a motorized delay stage. The pump pulses were chopped by a synchronized chopper at 500 Hz and the induced absorbance change was calculated with two adjacent probe pulses (pump-blocked and pump-unblocked). All experiments were performed at room temperature.

Supplemental Figure and Table

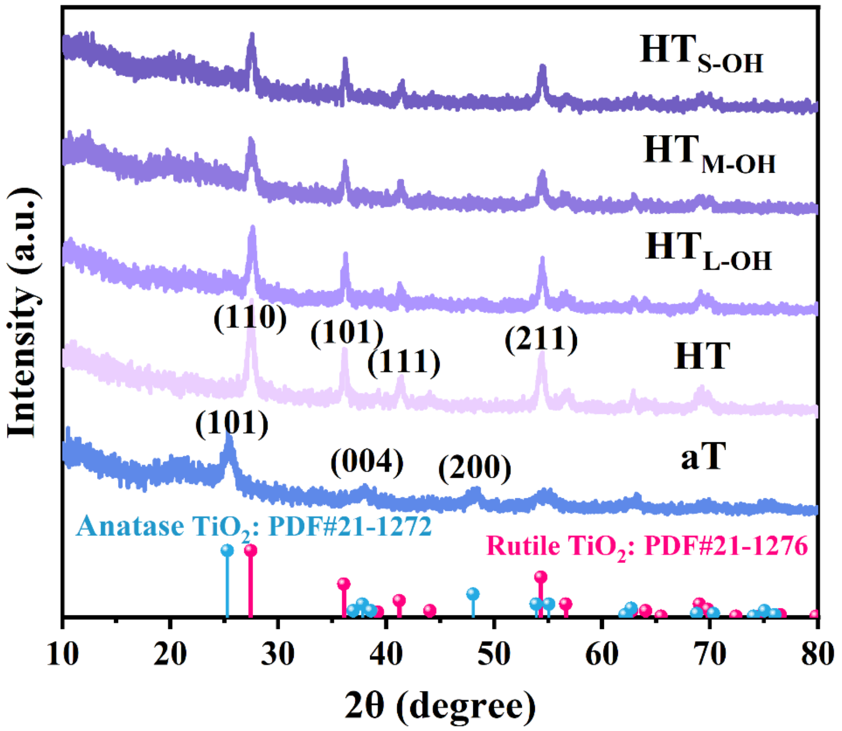

Figure S1. XRD patterns of aT, HT and $HT_{X\text{-}OH}$.

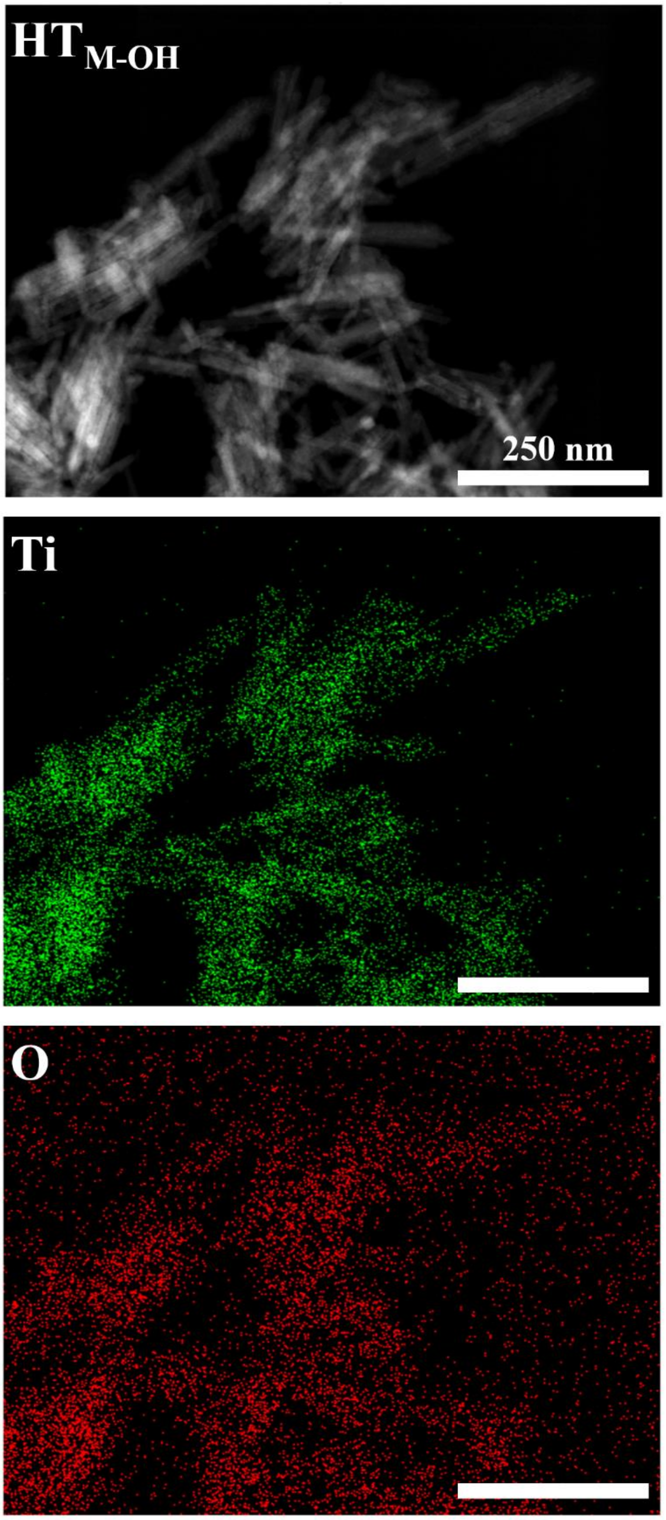

Figure S2. EDS mapping images of HT$_{M\text{-}OH}$.

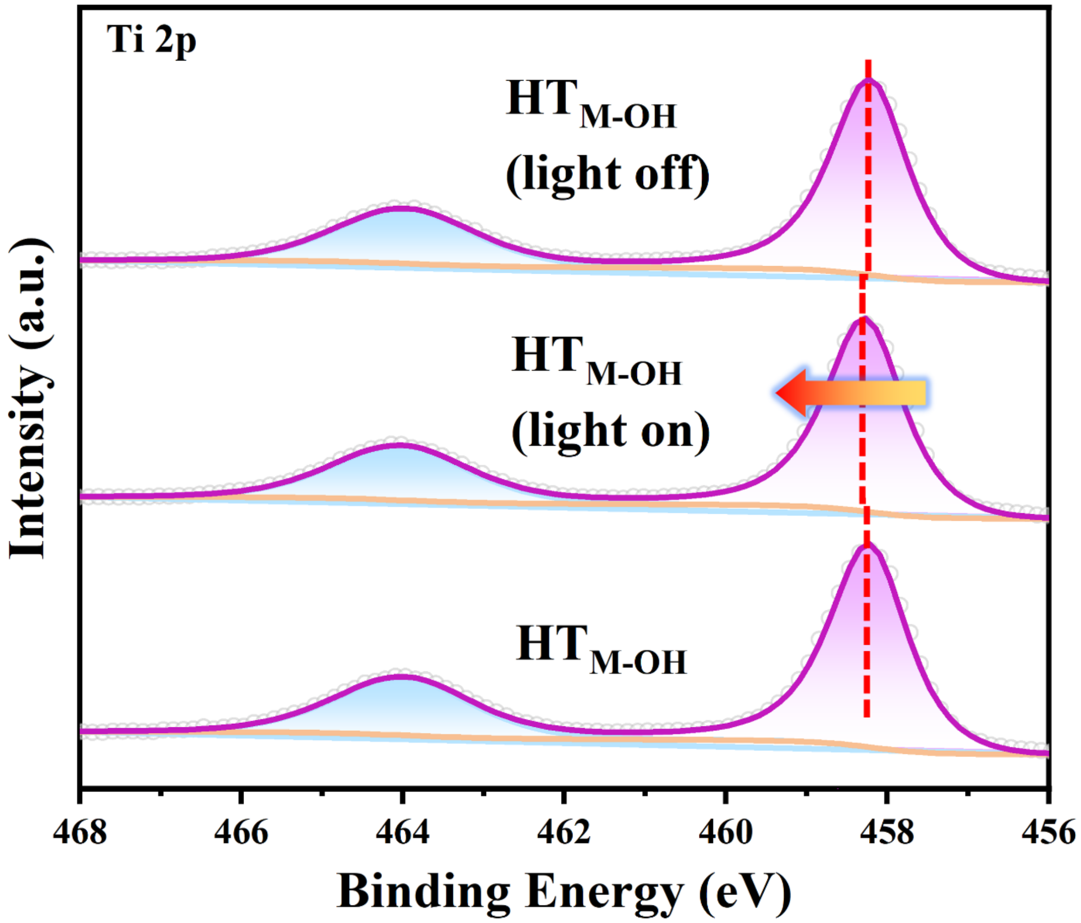

Figure S3. In-situ XPS spectra of Ti 2p.

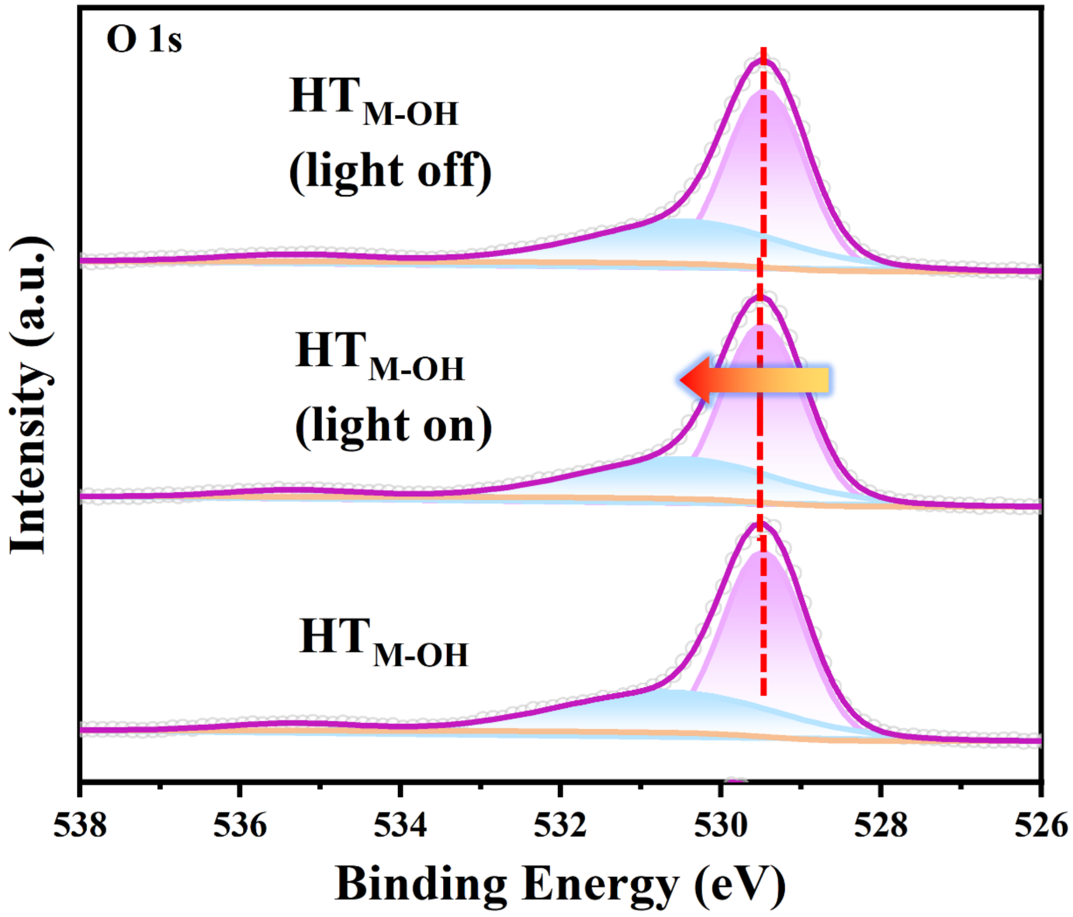

Figure S4. In-situ XPS spectra of O 1s.

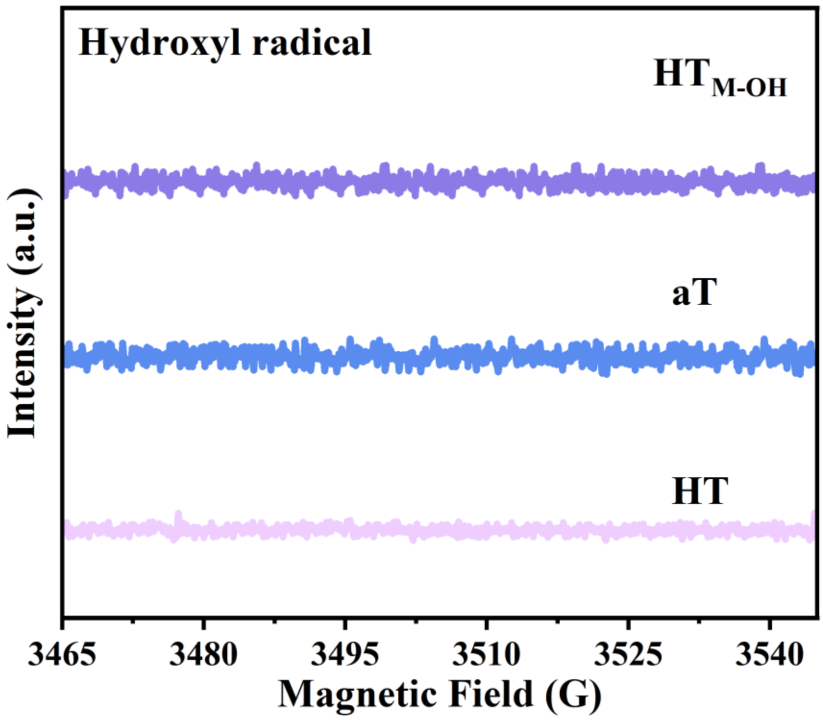

Figure S5. ESR signals of HT, aT and $HT_{M\text{-}OH}$ in aqueous dispersion for DMPO-hydroxyl radical under dark condition.

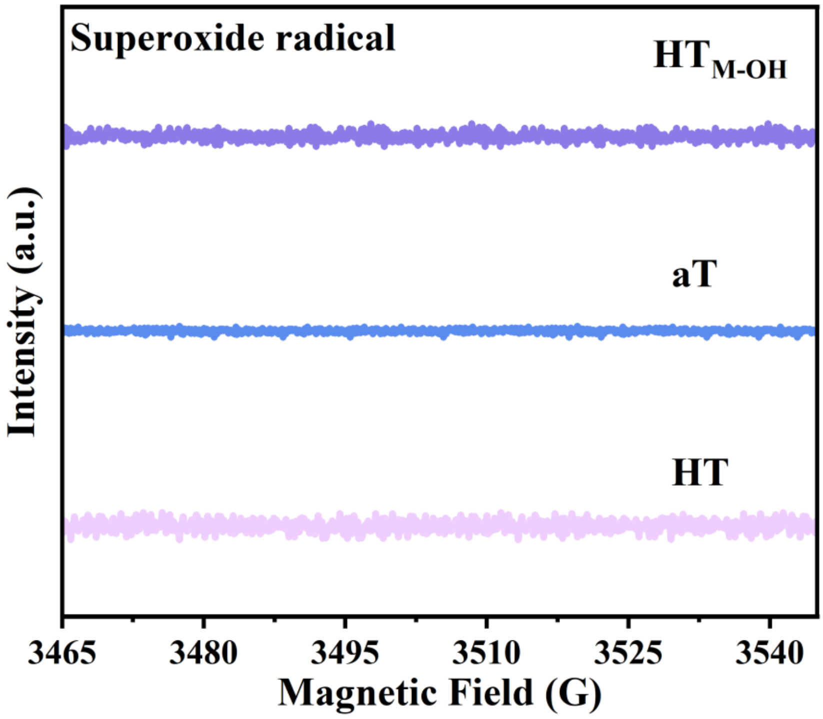

Figure S6. ESR signals of HT, aT and $HT_{M\text{-}OH}$ in methanol dispersion for DMPO-superoxide radical under dark condition.

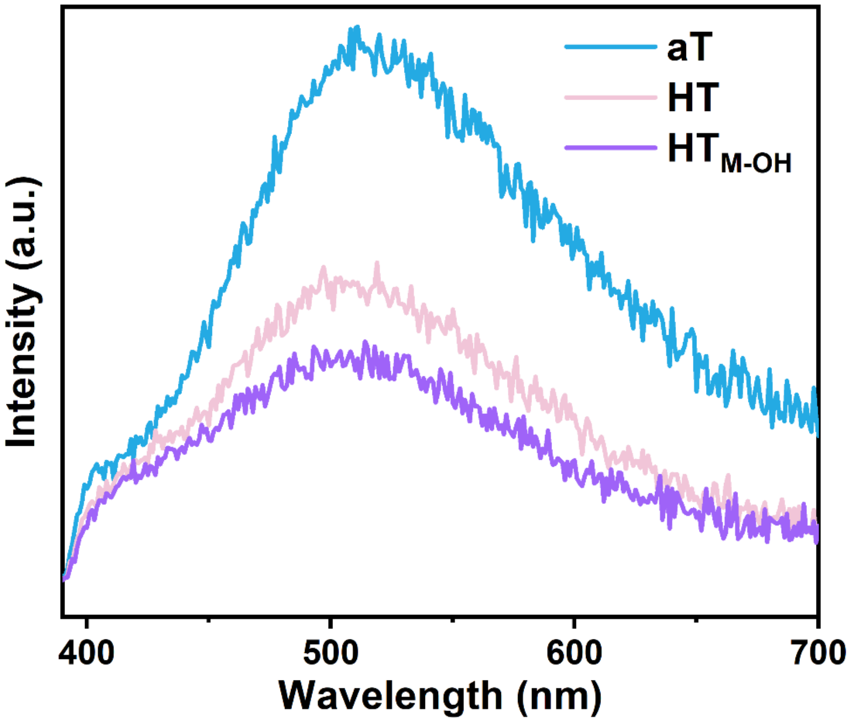

Figure S7. Photoluminescence emission spectrum of HT, aT and $HT_{M\text{-}OH}$ catalysts under the condition of fixed 365 nm excitation light.

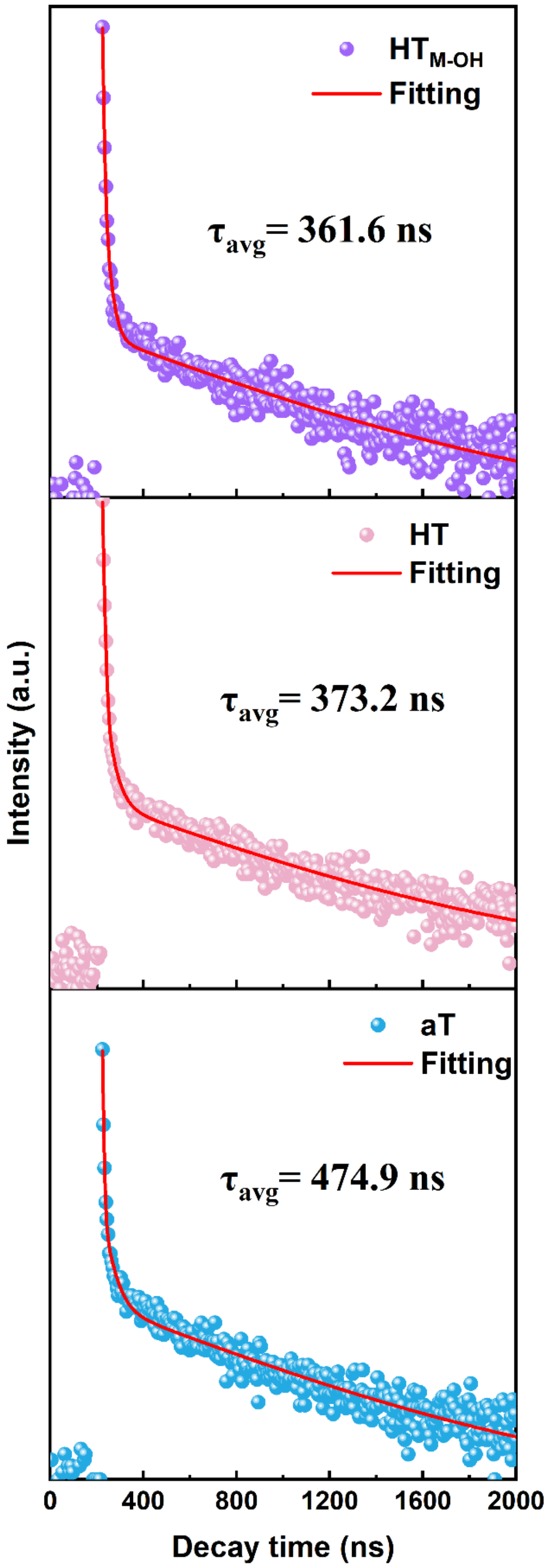

Figure S8. Time-resolved fluorescence spectra of of HT, aT and $HT_{M\text{-}OH}$ catalysts.

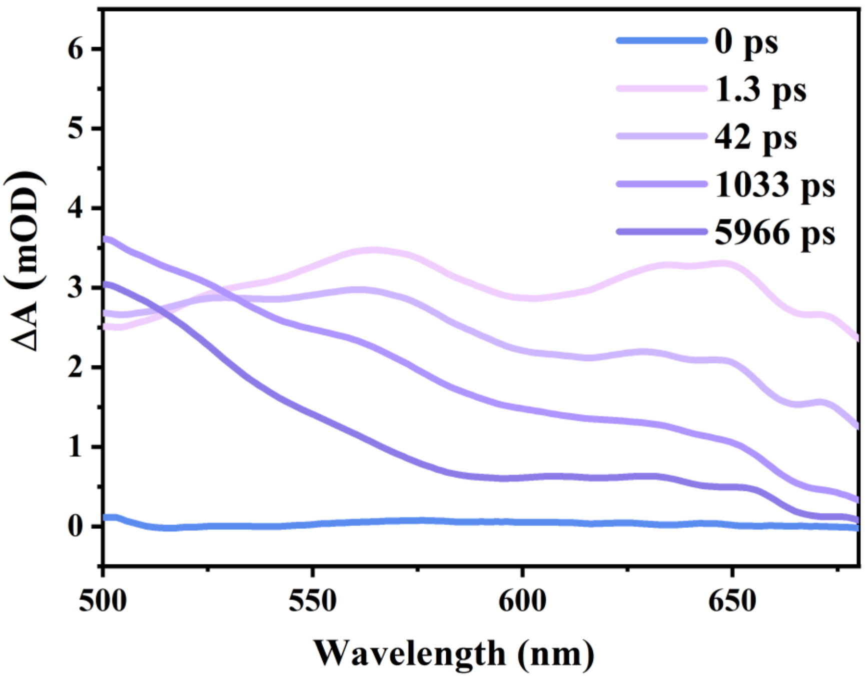

Figure S9. Transient absorption spectra of HT.

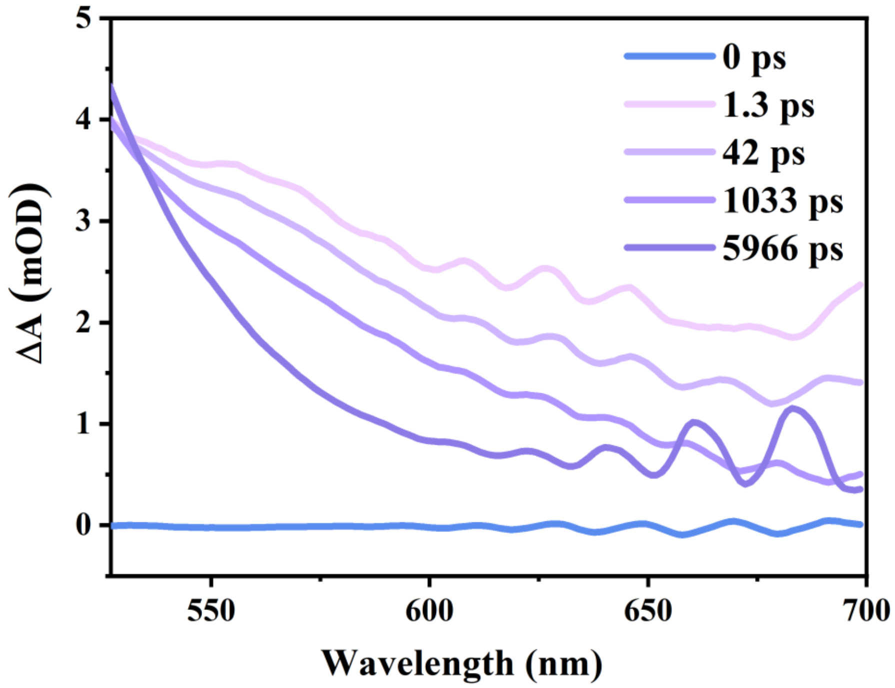

Figure S10. Transient absorption spectra of aT.

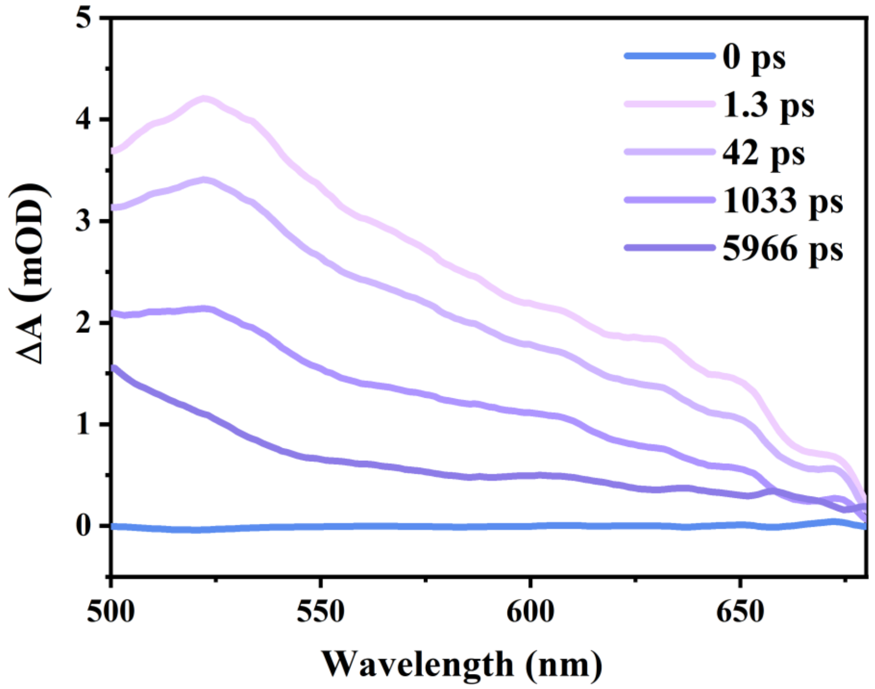

Figure S11. Transient absorption spectra of $HT_{M\text{-}OH}$.

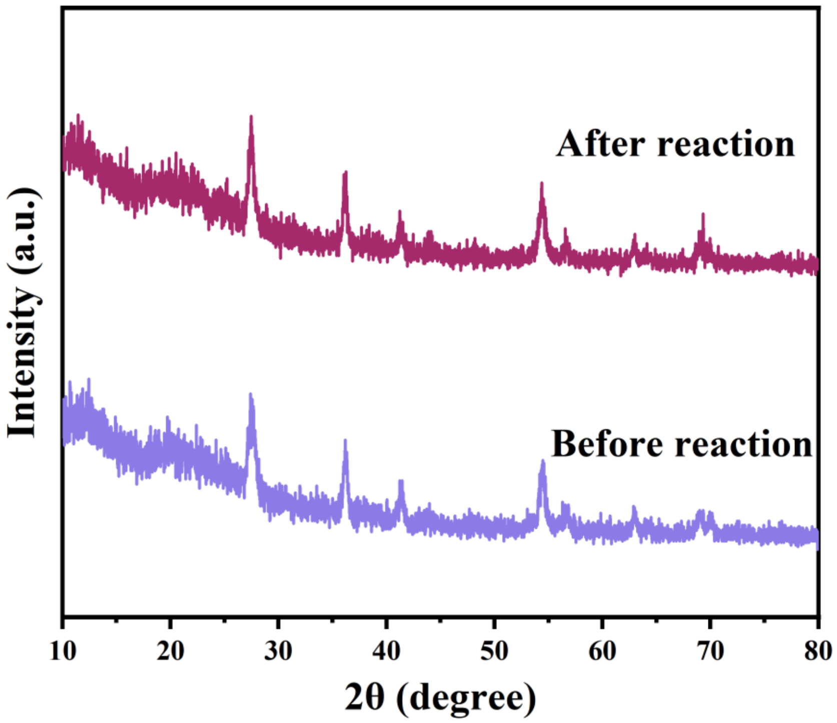

Figure S12. XRD patterns of HT_{M-OH} before and after reaction.

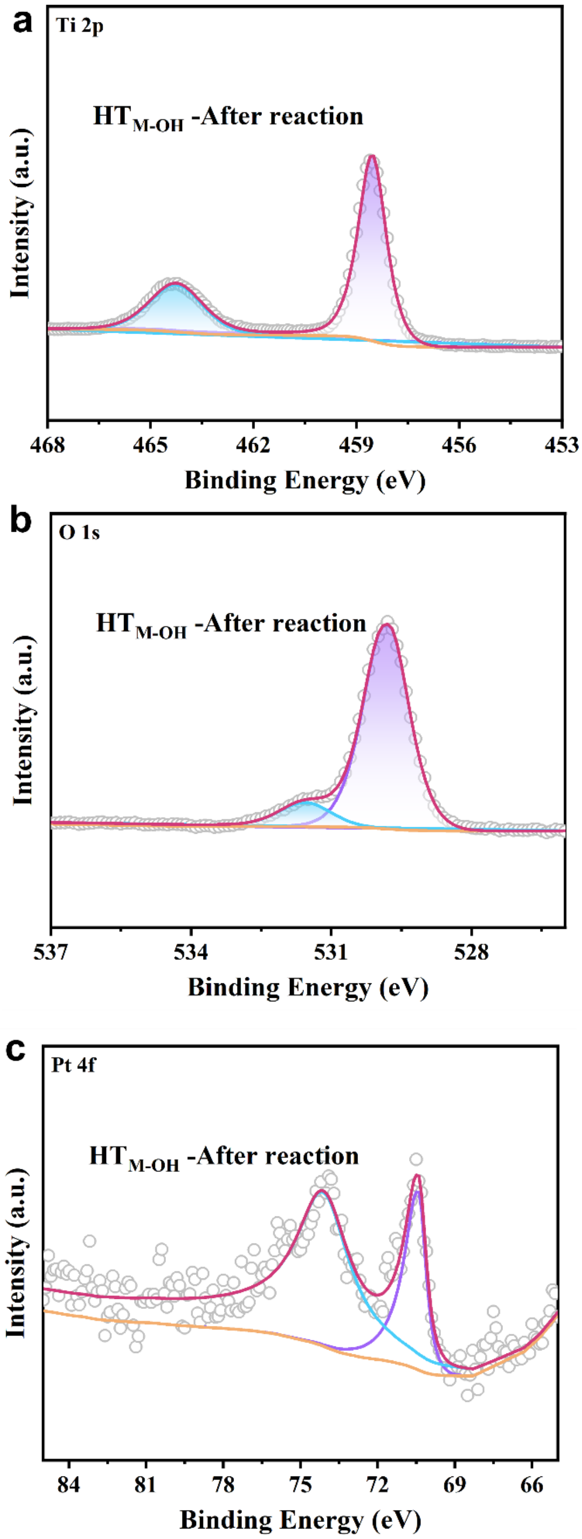

Figure S13. XPS spectra of $HT_{M\text{-}OH}$-after reaction.

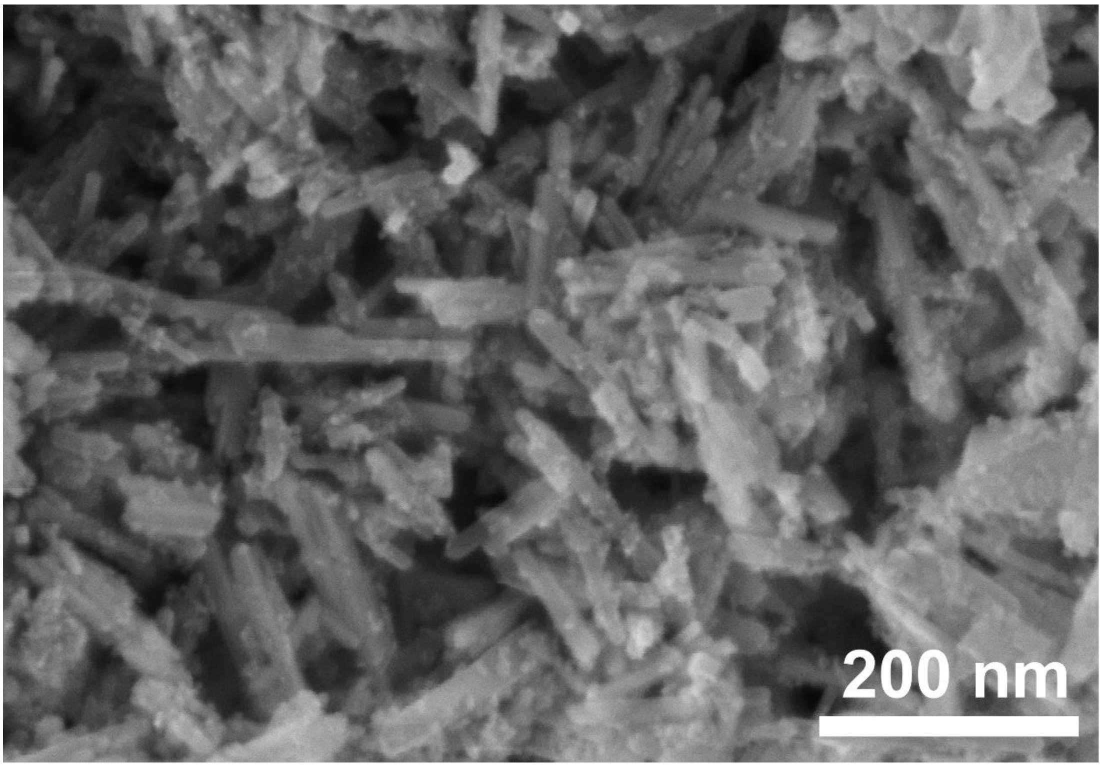

Figure S14. SEM image of $HT_{M\text{-}OH}$-after reaction.

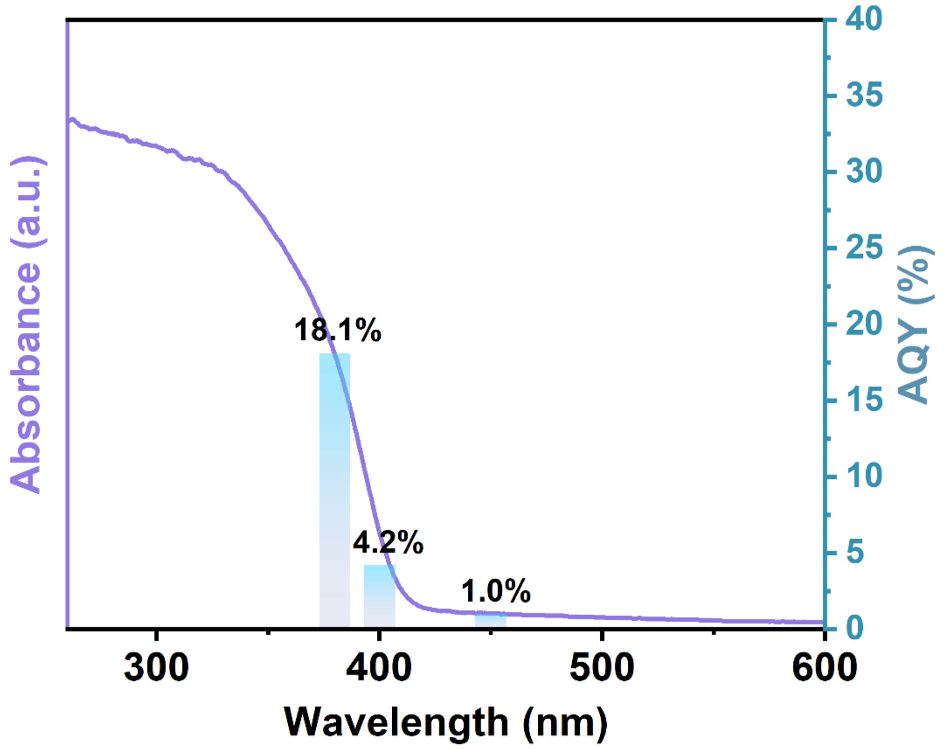

Figure S15. Apparent quantum efficiency of $HT_{M\text{-}OH}$.

Table S1. Fitted lifetimes of the normalized transient absorption kinetics from Fig.4.

	A_1	τ_1 (ps)	A_2	τ_2 (ps)	A_3	τ_3 (ps)	τ_{avg} (ps)
HT	13.9%	0.28	5.4%	4.9	80.6%	4537.9	3657.8
aT	72.6%	0.30	6.3%	957.7	21.0%	8664.5	1880.1
$HT_{M\text{-}OH}$	12.7%	3.37	14.8%	478.3	72.3%	5275.3	3885.2

Table S2. Summary of the hydrogen evolution of TiO_2-based materials.

Catalysts	PHE (mmol g^{-1} h^{-1})	Condition	Ref.
**HT_{M-OH}**	**34.35**	**MeOH (20 vol%)** 300 **W Xe lamp**	**This work**
a-Ti/H-Ti-2	29.63	MeOH (20 vol%) 300 W Xe lamp	[7]
P25-AP-1	29.14	MeOH (50 vol%) 300 W Xe lamp	[8]
$RuMoS_{2+x}/TiO_2$-7	26.49	TEOA (10 vol%, pH=11) 365nm LED lamps	[9]
Co—RuO_x/TiO_2	20.20	TEOA (10 vol%) 300 W Xe lamp	[10]
PF3T@Au-TiO_2	18.58	water: MeOH: triethylamine (1:1:1) 350 W Xe-lamp>400nm	[11]
TiO_2/Ti_3C_2/CNS	15.29	TEOA (10 vol%) > 400 nm	[12]
$ReSe_{2+x}/TiO_2$	12.49	EtOH (25 vol%) 300 W Xe lamp	[13]
$Re_{0.92}Mo_{0.08}S_2/TiO_2$	10.56	Lactic acid(20 vol%) 365nm LED lamps	[14]
Ru_1/TiNS	8.95	TEOA (20 vol%) 300 W Xe lamp	[15]
Au/TiO_2	8.50	EtOH (10 vol%) 300 W Xe lamp	[16]
PCTI	8.15	MeOH (20 vol%) 300 W Xe lamp	[17]
TiO_2/Au@ReS_{2+x}	6.01	TEOA (10 vol%) 365nm LED lamps	[18]
$Ni_6(SCH_2Ph)_{12}/TiO_2$	5.60	MeOH (20 vol%) 300 W Xe lamp	[19]
Co_2P/PC-b-TiO_2	1.53	TEOA (10 vol%) 300 W Xe lamp	[20]

References

[1] G. Kresse, J. Hafner, *Phys. Rev. B* **1993**, 47, 558.
[2] G. Kresse, J. Furthmüller, *Phys. Rev. B* **1996**, 54, 11169.
[3] P. E. Blöchl, *Phys. Rev. B* **1994**, 50, 17953.
[4] G. Kresse, D. Joubert, *Phys. Rev. B* **1999**, 59, 1758.
[5] J. P. Perdew, K. Burke, M. Ernzerhof, *Phys. Rev. Lett.* **1996**, 77, 3865.
[6] S. Grimme, *J. Comput. Chem.* **2006**, 27, 1787.
[7] X. Ruan, X. Cui, Y. Cui, X. Fan, Z. Li, T. Xie, K. Ba, G. Jia, H. Zhang, L. Zhang, W. Zhang, X. Zhao, J. Leng, S. Jin, D. J. Singh, W. Zheng, *Adv. Energy Mater.* **2022**, 12, 2200298.
[8] R. Wang, G. Che, C. Wang, C. Liu, B. Liu, B. Ohtani, Y. Liu, X. Zhang, *ACS Catal.* **2022**, 12, 12206.
[9] W. Zhong, D. Gao, P. Wang, X. Wang, H. Yu, *Appl. Catal. B* **2022**, 319, 121910.
[10] J. Shen, C. Luo, S. Qiao, Y. Chen, K. Fu, J. Xu, J. Pei, Y. Tang, X. Zhang, H. Tang, H. Zhang, C. Liu, *Adv. Funct. Mater.* **2024**, 34, 2309056.
[11] J.-C. Kao, D. Bhalothia, Z.-X. Wang, H.-W. Lin, F.-G. Tseng, L.-Y. Ting, H.-H. Chou, Y.-C. Lo, J.-P. Chou, T.-Y. Chen, *Small* **2023**, 19, 2303391.
[12] H. Zeng, Z. Li, G. Li, X. Cui, M. Jin, T. Xie, L. Liu, M. Jiang, X. Zhong, Y. Zhang, H. Zhang, K. Ba, Z. Yan, Y. Wang, S. Song, K. Huang, S. Feng, *Adv. Energy Mater.* **2022**, 12, 2102765.
[13] D. Gao, H. Long, X. Wang, J. Yu, H. Yu, *Adv. Funct. Mater.* **2023**, 33, 2209994.
[14] J. Xu, W. Zhong, X. Zhang, X. Wang, X. Hong, H. Yu, *Small* **2023**, 19, 2303960.
[15] J. Li, D. Yi, F. Zhan, B. Zhou, D. Gao, D. Guo, S. Liu, X. Wang, J. Yao, *Appl. Catal. B* **2020**, 271, 118925.
[16] M. Torras, P. Molet, L. Soler, J. Llorca, A. Roig, A. Mihi, *Adv. Energy Mater.* **2022**, 12, 2103733.
[17] H. S. Moon, K.-C. Hsiao, M.-C. Wu, Y. Yun, Y.-J. Hsu, K. Yong, *Adv. Mater.* **2023**, 35, 2200172.
[18] W. Zhong, J. Xu, X. Zhang, J. Zhang, X. Wang, H. Yu, *Adv. Funct. Mater.* **2023**, 33, 2302325.
[19] F. Tian, J. Chen, F. Chen, Y. Liu, Y. Xu, R. Chen, *Appl. Catal. B* **2021**, 292, 120158.
[20] L. Chen, X.-L. Song, J.-T. Ren, Z.-Y. Yuan, *Appl. Catal. B* **2022**, 315, 121546.
